# Supplementary material for: Nascent ribosomal RNA act as surfactant that suppresses growth of fibrillar centers in nucleolus
Source: Commun Biol. 2023 Nov 7;6:1129. doi: 10.1038/s42003-023-05519-1 (PMC10630424; doi:10.1038/s42003-023-05519-1)
Supplement: Supplementary file 2 — Supplementary Information [file 42003_2023_5519_MOESM2_ESM.pdf]

Supplementary Information: Nascent ribosomal RNA act as surfactant that suppresses growth of fibrillar centers in nucleolus

Tetsuya Yamamoto, Tomohiro Yamazaki, Kensuke Ninomiya, and Tetsuro Hirose

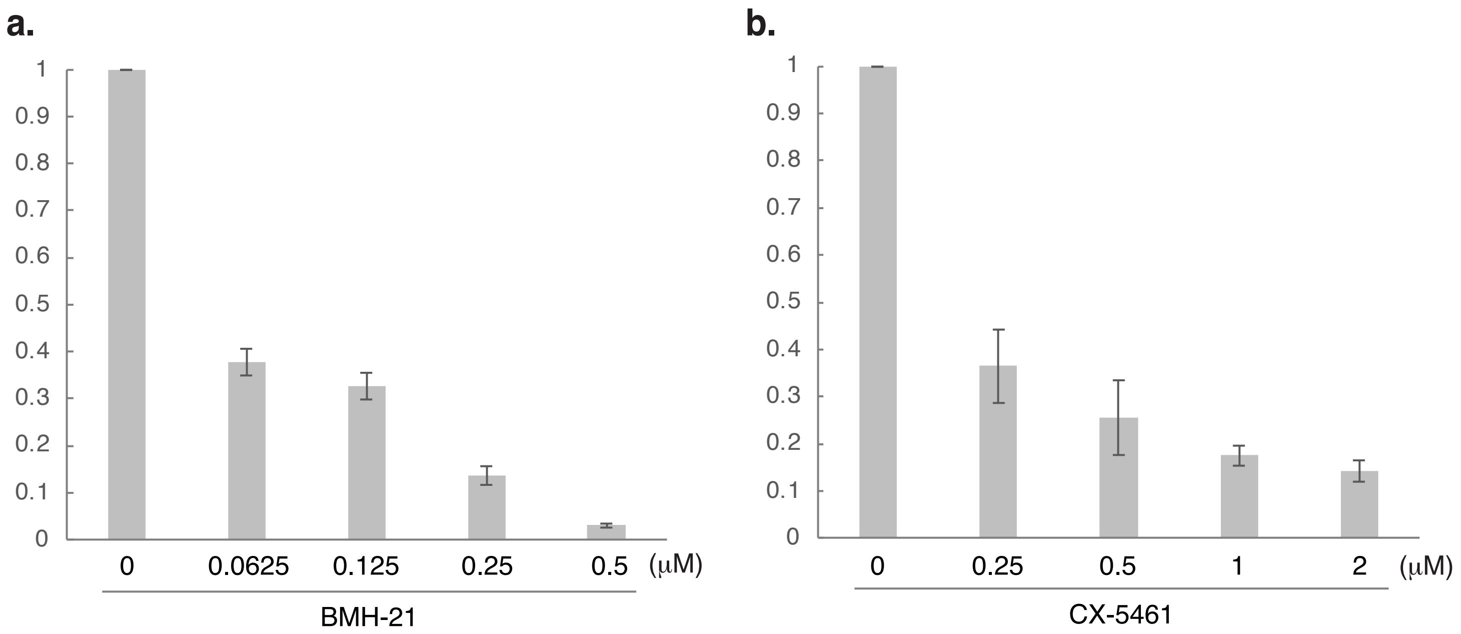

Figure S1 BMH-21 and CX-5461 treatment reduce pre-rRNA expression levels in a dose-dependent manner.

a. Quantification of pre-rRNA expression levels by RT-qPCR in BMH-21-untreated and -treated conditions. Data are represented as mean ± SD (n =3). **b.** Quantification of pre-rRNA expression levels by RT-qPCR in CX-5461-untreated and -treated conditions. Data are represented as mean ± SD (n =3).

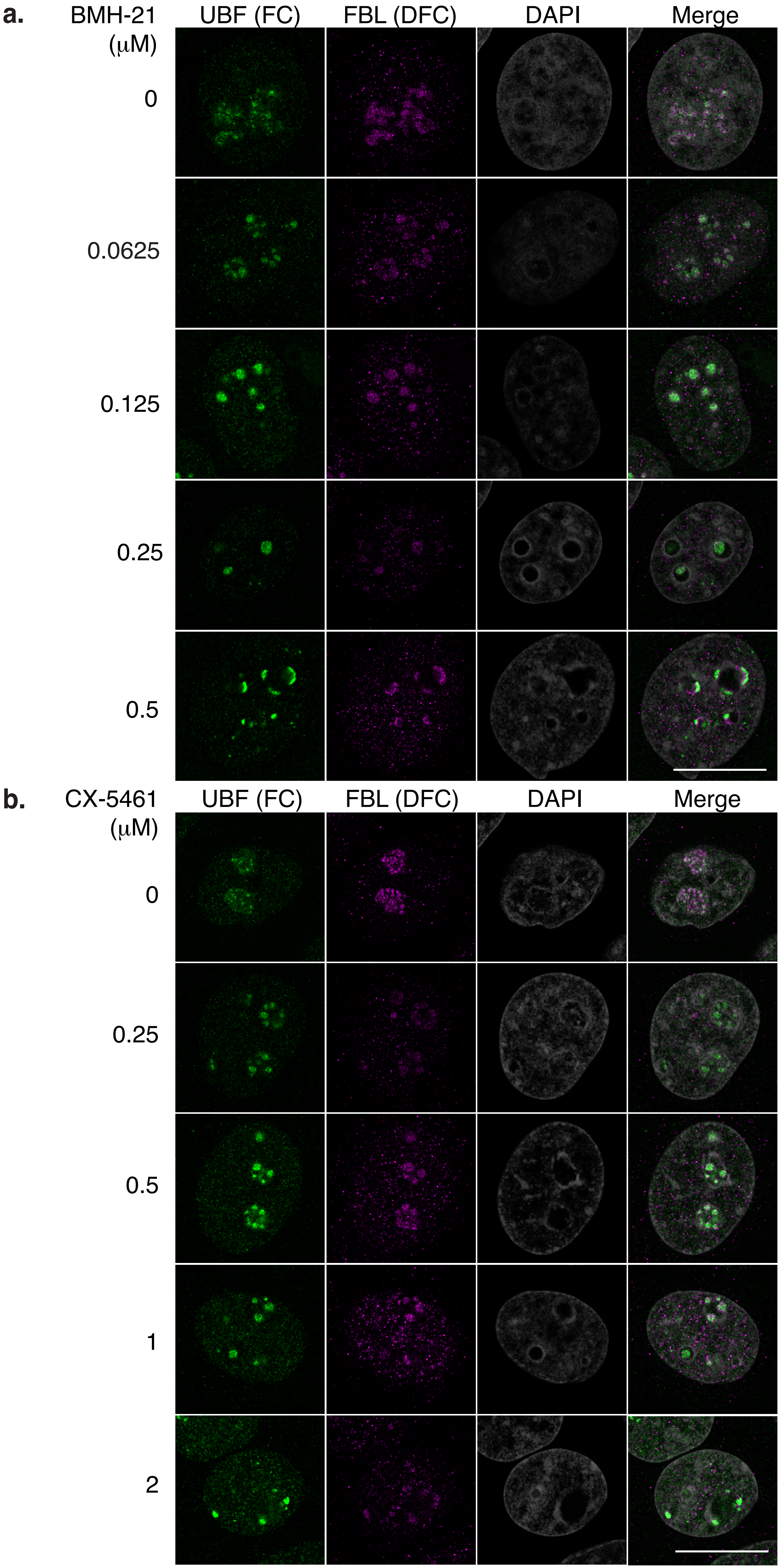

a.
BMH-21 (μM)
UBF (FC)
FBL (DFC)
DAPI
Merge
0
0.0625
0.125
0.25
0.5
b.
CX-5461 (μM)
UBF (FC)
FBL (DFC)
DAPI
Merge
0
0.25
0.5
1
2

Figure S2 FCs and DFCs in the BMH-21 and CX-5461-untreated and -treated cells. (**a** and **b**) Immunofluorescence of UBF (FC) and FBL (DFC) in HeLa cells with or without BMH-21 (**a**) or CX-5461 (**b**) treatments. Scale bar, 10 μm.

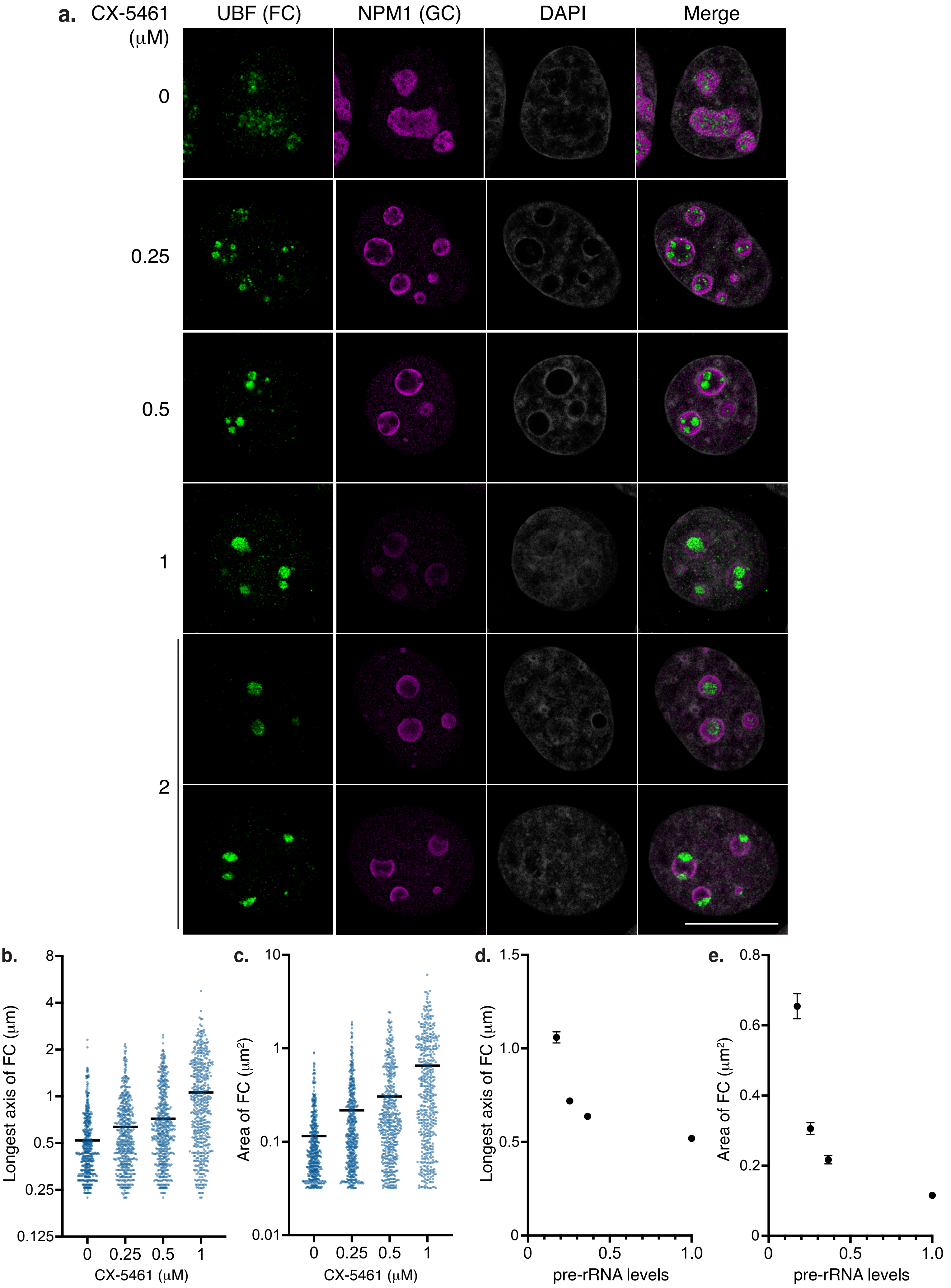
a.
CX-5461 (μM)
UBF (FC)
NPM1 (GC)
DAPI
Merge
0
0.25
0.5
1
2
b.
Longest axis of FC (μm)
8
4
2
1
0.5
0.25
0.125
0
0.25
0.5
1
CX-5461 (μM)
c.
Area of FC (μm²)
10
1
0.1
0.01
0
0.25
0.5
1
CX-5461 (μM)
d.
Longest axis of FC (μm)
1.5
1.0
0.5
0
0
0.5
1.0
pre-rRNA levels
e.
Area of FC (μm²)
0.8
0.6
0.4
0.2
0
0
0.5
1.0
pre-rRNA levels

Figure S3 Mild Pol I inhibition by CX-5461 increases the size of FCs.

a. Immunofluorescence of UBF (FC) and NPM1 (GC) in HeLa cells with or without CX-5461 treatments. In the cells treated with 2 μM CX-5461, the cells with large granules (upper) or nucleolar caps (lower) were observed. Scale bar, 10 μm. (**b** and **c**) Quantification of longest axis (**b**) and area (**c**) of the FCs in cells under indicated conditions. Each scatter dot plot shows the mean (black line). Dots indicate all points of quantified data (n = 500). Mean longest axes of the FCs is shown below: 0 μM: 0.5196 μm, 0.25 μM: 0.6367 μm, 0.5 μM: 0.7192 μm, 1 μM: 1.059 μm. Mean areas of the FCs are shown below: 0 μM: 0.1155 μm^2, 0. 25 μM: 0.2171 μm^2, 0.5 μM: 0.3058 μm^2, 1 μM: 0.6544 μm^2. Statistical analyses using Kruskal-Wallis test with Dunn's multiple comparison test were performed and the results are shown as follows. (**b**) 0 μM vs 0.25 μM: $P < 0.0001$, 0 μM vs 0.5 μM: $P < 0.0001$, 0 μM vs 1 μM: $P < 0.0001$, 0.25 μM vs 0.5 μM: $P = 0.0211$, 0.25 μM vs 1 μM: $P < 0.0001$, 0.5 μM vs 1 μM: $P < 0.0001$. (**c**) 0 μM vs 0.25 μM: $P < 0.0001$, 0 μM vs 0.5 μM: $P < 0.0001$, 0 μM vs 1 μM: $P < 0.0001$, 0.25 μM vs 0.5 μM: $P = 0.0012$, 0.25 μM vs 1 μM: $P < 0.0001$, 0.5 μM vs 1 μM: $P < 0.0001$. (**d** and **e**) Graphs showing the mean longest axis (**d**) and area (**e**) of the FCs with SEM vs pre-rRNA expression levels. The pre-rRNA expression level in untreated cells is defined as 1.

a. BMH-21 (-)

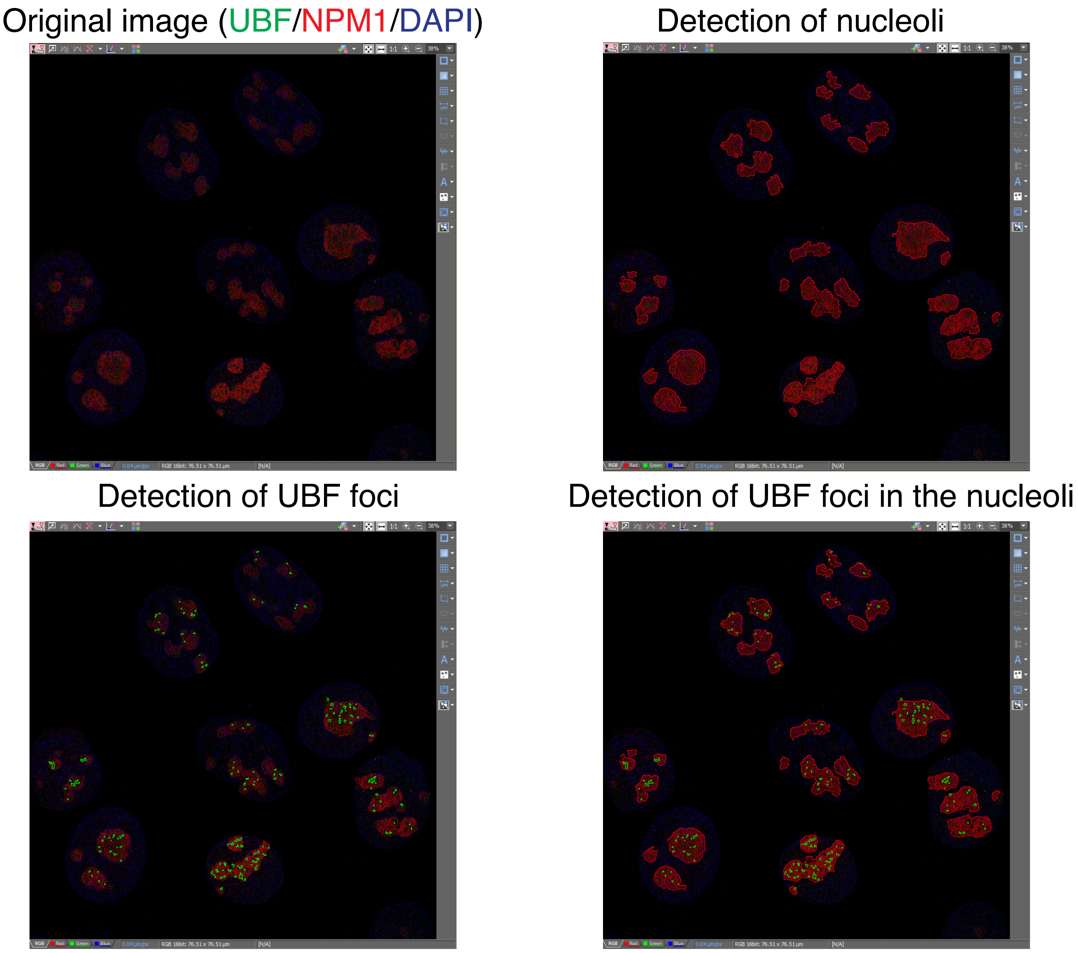

b. BMH-21 (0.125 μM)

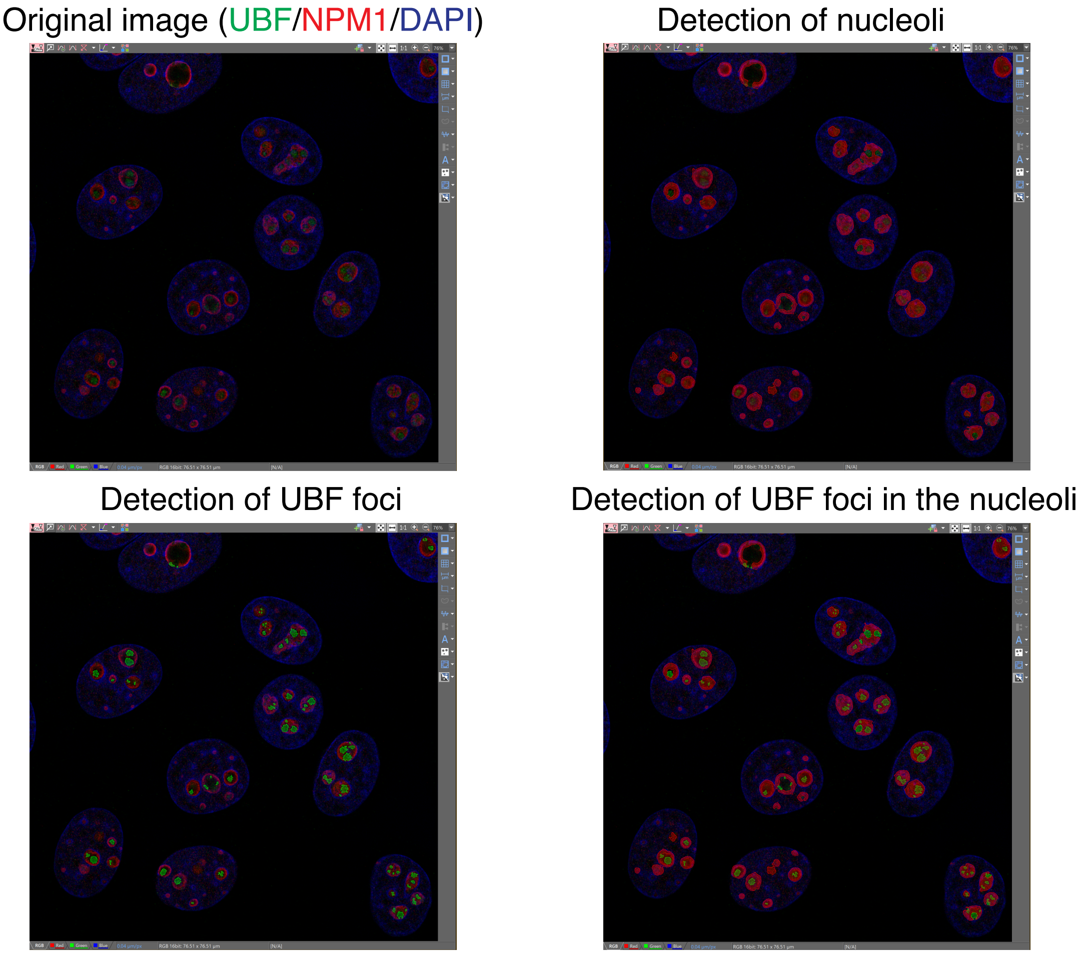

Figure S4 Quantification of the FCs in the nucleoli

(**a** and **b**) Screenshots of detections of FCs and GCs by NIS Elements Advanced Research software (NIKON) are shown. Green circles indicate UBF foci, and red circles indicate nucleoli labeled by NPM1 antibodies. UBF foci detected in the nucleoli (lower right) were quantified. Data for quantification of BMH-21-untreated (**a**) and -treated (0.125 mM) (**b**) cells are shown.

Supplementary Table 1: List of symbols

Symbol	Meaning	Estimate	Suppl. Ref.
r_{in}	Radius of FC	~ 100 nm	[1]
r_{ex}	Radius of FC/DFC	~ 200 nm	[1]
V_{m}	Sum of FC volume in a nucleolus	–	
τ_{pr}	Mean processing time	–	
σ_{in}	Nascent pre-rRNA surface density	–	
N_{r}	Number of units in pre-rRNA terminal region	33	[1]
b	Length of pre-rRNA unit	4 nm	[2]
F_{d}	Free energy per DFC layer	–	
f_{ela}	Elastic free energy density	–	
f_{mix}	Mixing free energy density	–	
f_{int}	Interaction free energy density	–	
f_{bnd}	Binding free energy density	–	
F	Free energy of a nucleolus	–	
γ_{in}	FC/DFC interfacial energy per area	–	
γ_{ex}	FC/GC interfacial energy per area –		
γ_{pr}	Interfacial energy per RBP	0.3 mJ/m^2	[3,4]
α_{pr}	RBP occupany of RNA unit	–	
ϕ_{pr}	Freely diffusing RBP volume fraction	–	
ϕ_{r}	Nascent pre-rRNA volume fraction	–	
ϵ	RBP-pre-rRNA binding energy	< -10	
χ	Magnitude of RBP-RBP interaction	> 10	
$\mu_{\mathrm{pr}}/(k_{\mathrm{B}}T)$	RBP chemical potential	-10	[5]
n_{ps}	Pol I occupancy at TSS	–	
$k_{\mathrm{on}}\rho$	Pol I binding rate to TSS	0.02 s^{-1}	[6]
k_{off}	Pol I unbinding rate from TSS	0.1 s^{-1}	[6]
ρ	Pol I concentration in FC	–	
n_{pe}	Number of Pol I with pre-rRNA terminal region	–	
n_{p0}	Number of Pol I without pre-rRNA terminal region	–	
τ_0	Mean Pol I elongation time (after cleavage)	–	
N_{a}	Copy number of active rDNA units	300	[1]
N_{pol}	Total number of Pol I in a nucleolus	7500	[1]
r	Distance from FC center	–	
$\Delta\Pi_{\parallel}$	Lateral surface pressure	–	

Supplementary Table 2: The values of the parameters used to estimate the diffusion length λ_{d}. The diffusion constant of a RNA unit is estimated as $D_{\mathrm{s}} \sim (k_{\mathrm{B}}T)/(4\pi\epsilon_{\mathrm{w}}b) \sim 8 \times 10^{-11}$ m^2/s and the time to synthesize a RNA unit is estimated as $\tau_{\mathrm{s}} = b/v_{\mathrm{e}} \sim 0.12$ s by using the values listed in this table. We estimated b from the persistence length of single-stranded DNA.

Parameter	Meaning	Values	Ref.
b	Length of RNA unit	4 nm $\approx$ 12 b	[2]
η_{w}	Water viscosity	1 mPa $\cdot$ s	
v_{e}	Elongation rate	6 kb/min	[7,8]
T	Absolute temperature	300 K	

Supplementary Note 1; Minimum of free energy

Free energy

The free energy of the system has the form

$$\frac{F}{V_{\mathrm{m}}} = \frac{3}{4\pi r_{\mathrm{in}}^3}\left[F_{\mathrm{d}} + 4\pi r_{\mathrm{in}}^2 \gamma_{\mathrm{in}} + 4\pi r_{\mathrm{ex}}^2 \gamma_{\mathrm{ex}}\right]. \tag{S1}$$

F_{d} is the free energy of a DFC layer and is a functional of the occupancy α_{p} of the fibrillarin-binding region (FBR) of nascent pre-rRNA, the volume fraction ϕ_{p} of fibrillarin (FBL) RNA-binding proteins (RBPs), and the volume fraction ϕ_{r} of the nascent pre-rRNA units (see below). γ_{in} is the interfacial tensions at the FC-DFC interface and γ_{ex} is the interfacial tension at the DFC-GC interface. r_{in} is the distance between the center of FC and the FC-DFC interface. r_{ex} is the distance between the center of FC and the DFC-GC interface.

The free energy of a DFC layer is written in the form

$$F_{\mathrm{d}} = \int_{r_{\mathrm{in}}}^{r_{\mathrm{ex}}} \frac{4\pi r^2 dr}{b^3} f_{\mathrm{d}}, \tag{S2}$$

where f_{d} is the free energy density in the DFC layer and r is the radical coordinate from the center of the FC. b is the length of a pre-rRNA unit. The free energy density f_{d} is composed of 4 contributions

$$f_{\mathrm{d}} = f_{\mathrm{ela}} + f_{\mathrm{mix}} + f_{\mathrm{int}} + f_{\mathrm{bnd}} - \mu_{\mathrm{p}}(\phi_{\mathrm{p}} + \alpha_{\mathrm{p}}\phi_{\mathrm{r}}) + \Pi_{\mathrm{ex}} b^3, \tag{S3}$$

where f_{ela} is the elastic free energy density of nascent RNA transcripts, f_{mix} is the free energy density due to the mixing entropy of RBPs and solvent molecules, f_{int} is the free energy density due to the interactions between RBPs, and f_{bnd} is the free energy density due to the binding of RBPs to nascent RNA transcripts. μ_{p} is the chemical potential of RBPs and Π_{ex} is the external pressure. The external radius r_{ex} and the volume fraction ϕ_{r}

of nascent pre-rRNA have the relationship

$$\int_{r_{\rm in}}^{r_{\rm ex}} dr\, 4\pi r^2 \phi_{\rm r} = 4\pi r_{\rm in}^2 \sigma_{\rm in} N_{\rm r}. \tag{S4}$$

The elastic free energy density has the form

$$\frac{f_{\rm ela}}{k_{\rm B}T} = \frac{3}{2}\frac{b^4\sigma^2(r)}{\phi_{\rm r}}, \tag{S5}$$

where $\sigma(r) = \sigma_{\rm in} r_{\rm in}^2/r^2$ for the spherical geometry and $\sigma(r) = \sigma_{\rm in}$ for the planer geometry. $k_{\rm B}$ is the Boltzmann constant and T is the absolute temperature. The derivation of Supplementary Equation (S5) is shown in Supplementary Note 5.

The free energy due to the mixing entropy of RBPs and solvent molecules has the form

$$\frac{f_{\rm mix}}{k_{\rm B}T} = \phi_{\rm p}\log\phi_{\rm p} + (1-\phi_{\rm p}-(1+\alpha_{\rm p})\phi_{\rm r})\log(1-\phi_{\rm p}-(1+\alpha_{\rm p})\phi_{\rm r}). \tag{S6}$$

The free energy due to the interactions between RBPs has the form

$$\frac{f_{\rm int}}{k_{\rm B}T} = -\chi(\phi_{\rm p}+\alpha_{\rm p}\phi_{\rm r})^2, \tag{S7}$$

where χ is the interaction parameter that accounts for the attractive interaction between RBPs. For simplicity, we assumed that RBPs that are bound to nascent RNA transcripts are equivalent to RBPs that are freely diffusing in the DFC layer. The solvent molecules (water) have affinity to nascent RNA units rather than RPBs. We thus assume that solvent molecules and nascent pre-rRNA units are equal in terms of the interaction.

The free energy due to the binding of RBPs to nascent RNA transcripts has the form

$$\frac{f_{\rm bnd}}{k_{\rm B}T} = \phi_{\rm r}\left[\alpha_{\rm p}\log\alpha_{\rm p} + (1-\alpha_{\rm p})\log(1-\alpha_{\rm p}) + \epsilon\alpha_{\rm p}\right], \tag{S8}$$

where $-\epsilon k_{\rm B}T$ is the energy increase due to the binding of RBPs to nascent RNA transcripts.

For simplicity, we assumed that each nascent RNA unit has one binding site of RBPs.

Local equilibrium

With the variations $\alpha_{\rm p}(r) \to \alpha_{\rm p}(r) + \delta\alpha_{\rm p}(r)$, $\phi_{\rm p}(r) \to \phi_{\rm p}(r) + \delta\phi_{\rm p}(r)$, and $\phi_{\rm r}(r) \to \phi_{\rm r}(r) + \delta\phi_{\rm r}(r)$, the free energy changes as

$$\begin{aligned} \frac{\delta F}{V_{\rm m}} &= \frac{3}{4\pi r_{\rm in}^3}\int_{r_{\rm in}}^{r_{\rm ex}} dr\, \frac{4\pi r^2}{b^3}\left[\left(\frac{\delta f_{\rm d}}{\delta\alpha_{\rm p}(r)}\delta\alpha_{\rm p}(r) + \frac{\delta f_{\rm d}}{\delta\phi_{\rm p}(r)}\delta\phi_{\rm p}(r) + \frac{\delta f_{\rm d}}{\delta\phi_{\rm r}(r)}\delta\phi_{\rm r}(r)\right)\right] \\ &\quad +\delta r_{\rm ex}\frac{\partial}{\partial r_{\rm ex}}\left(\frac{F}{V_{\rm m}}\right). \end{aligned} \tag{S9}$$

Because the free energy density $f_{\rm d}$ does not include spatial derivatives of $\alpha_{\rm p}(r)$, $\phi_{\rm p}(r)$, and $\phi_{\rm r}(r)$, the functional derivatives of $f_{\rm d}$ with respect to $\alpha_{\rm pol}(r)$, $\phi_{\rm pol}(r)$, and $\phi_{\rm r}(r)$ can be replaced to the partial derivatives of $f_{\rm d}$ with respect to $\alpha_{\rm p}$, $\phi_{\rm p}$, and $\phi_{\rm r}$. The variation $\delta r_{\rm ex}$ of the external radius results from the variation $\delta\phi_{\rm r}(r)$ with the condition of eq. S4:

$$\delta r_{\rm ex} = -\frac{b^3}{4\pi r_{\rm ex}^2}\frac{1}{\phi_{\rm r}(r_{\rm ex})}\int_{r_{\rm in}}^{r_{\rm ex}} dr\,\frac{4\pi r^2}{b^3}\delta\phi_{\rm r}(r). \tag{S10}$$

At the minimum of the free energy (the local equilibrium), $\delta F = 0$ for any functions of $\delta\alpha_{\rm pol}(r)$, $\delta\phi_{\rm pol}(r)$, and $\delta\phi_{\rm r}(r)$:

$$\frac{\partial f_{\rm d}}{\partial\alpha_{\rm p}} = 0 \tag{S11}$$

$$\frac{\partial f_{\rm d}}{\partial\phi_{\rm p}} = 0 \tag{S12}$$

$$\frac{\partial f_{\rm d}}{\partial\phi_{\rm r}} = \mu_{\rm r} \tag{S13}$$

with

$$\mu_{\rm r} = \left.\frac{f_{\rm d}}{\phi_{\rm r}}\right|_{r\to r_{\rm ex}} + \left(\frac{2b^3\gamma_{\rm ex}}{r_{\rm ex}} + \Pi_{\rm ex}b^3\right)\frac{1}{\phi_{\rm r}(r_{\rm ex})}. \tag{S14}$$

By using Supplementary Equations (S3) − (S8), Supplementary equation (S12) is rewritten in the form

$$\frac{\mu_{\rm p}}{k_{\rm B}T} = \log\phi_{\rm p} - \log(1-\phi_{\rm p}-(1+\alpha_{\rm p})\phi_{\rm r}) - 2\chi(\phi_{\rm p}+\alpha_{\rm p}\phi_{\rm r}). \tag{S15}$$

Supplementary Equation (S15) suggests that the chemical potential of RBPs is uniform in the DFC layer. By using Supplementary Equations (S3) - (S8), Supplementary Equation (S11) is rewritten in the form

$$\log\alpha_{\rm p} - \log(1-\alpha_{\rm p}) + \epsilon - 1 - \log\phi_{\rm p} = 0, \tag{S16}$$

We eliminated $\mu_{\rm p}$ in Supplementary Equation (S11) by using Supplementary Equation (S15) to derive the form of Supplementary Equation (S16). By using Supplementary Equations (S3) - (S8), Supplementary Equation (S13) is rewritten in the form

$$\begin{aligned}\frac{\mu_{\rm r}}{k_{\rm B}T} &= -\frac{3}{2}\frac{(\sigma^2(r)b^2)^2}{\phi_{\rm r}^2} - (1+\alpha_{\rm p})\log(1-\phi_{\rm p}-(1+\alpha_{\rm p})\phi_{\rm r}) - (1+\alpha_{\rm p}) - 2\chi\alpha_{\rm p}(\phi_{\rm p}+\alpha_{\rm p}\phi_{\rm r}) \\ &\quad +\alpha_{\rm p}\log\alpha_{\rm p} + (1-\alpha_{\rm p})\log(1-\alpha_{\rm p}) + \epsilon\alpha_{\rm p} - \frac{\mu_{\rm p}}{k_{\rm B}T}\alpha_{\rm p}. \end{aligned} \tag{S17}$$

Supplementary Equation (S13) is integrated with respect to $\phi_{\rm r}$ as

$$f_{\rm d} - \mu_{\rm r}\phi_{\rm r} = -\Pi b^3, \tag{S18}$$

where $-\Pi b^3$ is the integral constant. By eliminating $\mu_{\rm r}$ from Supplementary Equation (S18) by using Supplementary Equation (S13), Supplementary Equation (S18) is rewritten in the form

$$\Pi b^3 = \phi_{\rm r}^2 \frac{\partial}{\partial \phi_{\rm r}}\left(\frac{f_{\rm d}}{\phi_{\rm r}}\right). \tag{S19}$$

The integral constant thus turns out to be the osmotic pressure. By using Supplementary

Equations (S3) - (S8), Supplementary Equation (S19) is rewritten in the form

$$\frac{\Pi b^3}{k_{\rm B}T} = -(1+\alpha_{\rm p})\phi_{\rm r} - \log(1-\phi_{\rm p}-(1+\alpha_{\rm p})\phi_{\rm r}) - \chi(\phi_{\rm p}+\alpha_{\rm p}\phi_{\rm r})^2 - \frac{3(\sigma(r)b^2)^2}{\phi_{\rm r}}, \quad \text{(S20)}$$

with

$$\sigma(r) = \frac{\sigma_{\rm in} r_{\rm in}^2}{r^2}. \quad \text{(S21)}$$

We eliminated $\mu_{\rm p}$ in Supplementary Equation (S19) by using Supplementary Equation (S15) to derive the form of Supplementary Equation (S20). So far, we derived the fact that the osmotic pressure is uniform in the DFC layer. The value of the osmotic pressure is determined by taking the limit $r \to r_{\rm ex}$ to Supplementary Equation (S18) as

$$\Pi = \Pi_{\rm ex} + \frac{2\gamma_{\rm ex}}{r_{\rm ex}}. \quad \text{(S22)}$$

Supplementary Note 2: Relaxation dynamics

In our theory, we take into account the non-equilibrium nature of the system via nascent pre-rRNAs produced at the surfaces of FCs by Pol I transcription. One may also think that the continuous addition of RNA units to nascent pre-rRNAs makes the conformations of their terminal regions different from those at the equilibrium. In a simple estimate, the local equilibrium approximation that uses the equilibrium chain conformations is effective in the length scales smaller than the diffusion length $\lambda_{\rm d}$ ($= \sqrt{D_{\rm s}\tau_{\rm s}}$, where $D_{\rm s}$ is the diffusion constant of a pre-rRNA unit and $\tau_{\rm s}$ is the elongation time to produce a pre-rRNA unit. The diffusion length $\lambda_{\rm d}$ is estimated as ~ 3 μm (see Supplementary Table 2) and is larger than the typical thickness, ~ 200 nm, of the DFC layer: the local equilibrium approximation is effective to treat the conformation of the pre-rRNAs in the DFC layer. The occupancy $\alpha_{\rm p}$,

the volume fractions ϕ_{p} and ϕ_{r} of RBPs and nascent pre-rRNA, and the radius r_{in} thus turn over towards the minimum of the free energy, eq. (2) in the main article.

Supplementary Note 3: Asymptotic solutions for planer geometry

The volume fractions ϕ_{r} and ϕ_{p} in the layer of nascent pre-rRNAs for the planer geometry are derived by using Supplementary Equations (S15), (S16), and (S20) for $\sigma(r) \to \sigma_{\mathrm{in}}$. We here derive the asymptotic forms of ϕ_{r} and ϕ_{p} for each regime.

Good and poor solvent regimes

For cases in which the interaction parameters χ is relatively small, the volume fraction ϕ_{r} of the FBRs of nascent pre-rRNAs and the volume fraction ϕ_{p} of RBPs are relatively small, $\phi_{\mathrm{r}} < 1$ and $\phi_{\mathrm{p}} < 1$, see Fig. 3 in the main article. With this approximation, Supplementary Equation (S15) has an approximate form

$$\frac{\mu_{\mathrm{p}}}{k_{\mathrm{B}}T} = \log \phi_{\mathrm{p}} \tag{S23}$$

Supplementary Equation (S23) is derived by expanding Supplementary Equation (S15) in a power series of ϕ_{r} and ϕ_{p} and neglecting higher order terms with respect to these volume fractions. By using Supplementary Equation (S23), the volume fraction of RBPs is derived in the form

$$\phi_{\mathrm{p0}} = \mathrm{e}^{\mu_{\mathrm{p}}/(k_{\mathrm{B}}T)}. \tag{S24}$$

By substituting Supplementary Equation (S24) into Supplementary Equation (S16), the occupancy of the FBRs of nascent pre-rRNAs is derived in the form

$$\alpha_{\mathrm{p0}} = \frac{\phi_{\mathrm{p0}} \mathrm{e}^{-(\epsilon-1)/(k_{\mathrm{B}}T)}}{1 + \phi_{\mathrm{p0}} \mathrm{e}^{-(\epsilon-1)/(k_{\mathrm{B}}T)}}. \tag{S25}$$

For $\phi_{\mathrm{r}} < 1$ and $\phi_{\mathrm{p}} < 1$, Supplementary Equation (S20) has an approximate form

$$\frac{\Pi_{\mathrm{ex}} b^3}{k_{\mathrm{B}}T} = -\frac{3(\sigma_{\mathrm{in}} b^2)^2}{\phi_{\mathrm{r}}} + (\chi_{\mathrm{s}} - \chi)\, \alpha_{\mathrm{p0}}^2 \phi_{\mathrm{r}}^2 + \frac{1}{3}(1 + \alpha_{\mathrm{p0}})^3 \phi_{\mathrm{r}}^3 \tag{S26}$$

with

$$\chi_{\mathrm{s}} = \frac{(1 + \alpha_{\mathrm{p0}})^2}{2\alpha_{\mathrm{p0}}^2}. \tag{S27}$$

Supplementary Equation (S26) is derived by expanding Supplementary Equation (S20) in a power series of ϕ_{p} and ϕ_{r} and neglecting higher order terms of these volume fractions. We assumed that ϕ_{p} is very small and neglected even the terms linear to ϕ_{p}. We also used the boundary condition $\Pi = \Pi_{\mathrm{ex}}$ for the planer geometry, see Supplementary Equation (S22) for $r_{\mathrm{ex}} \to \infty$. The osmotic pressure has three contributions: the contribution from the entropic elasticity of the RNP complexes (the first term of Suppplementary Equation (S26)), the contribution from the two-body interaction between the units of RNP complexes (the second term of Supplementary Equation (S26)), and the contribution from the three-body interactions between these units (the third term of Supplementary Equation (S26)). The two-body interaction between the units of RNA complexes is repulsive for $\chi < \chi_{\mathrm{s}}$ (the good solvent regime) and is attractive for $\chi > \chi_{\mathrm{s}}$ (the poor solvent regime). In the good solvent regime, $\chi < \chi_{\mathrm{s}}$, the first and second terms of Supplementary Equation (S26) dominate the third term of this equation and the volume fraction of the FBRs of nascent pre-rRNA thus

has the form

$$\phi_{\rm r0} = \left(\frac{3(\sigma_{\rm in}b^2)^2}{\alpha_{\rm p0}^2(\chi_{\rm s}-\chi)}\right)^{1/3} \tag{S28}$$

if there are not applied pressure to the DFC layer. In the poor solvent regime, $\chi > \chi_{\rm s}$, the second and third terms of Supplementary Equation (S26) dominates the third term of this equation and the volume fraction of the FBRs of nascent pre-rRNA has the form

$$\phi_{\rm r0} = \frac{3\alpha_{\rm p0}^2(\chi-\chi_{\rm s})}{(1+\alpha_{\rm p0})^3} \tag{S29}$$

Eqs. S28 and S29 correspond to the volume fraction of polymer units for the good and poor solvent conditions, as predicted by the Alexander model, respectively.

Melt regime

The volume fraction $\phi_{\rm r}$ of the FBRs of nascent pre-rRNAs increases with increasing the interaction parameter χ and, eventually, the RNP complexes occupy most volume of the DFC layer, $\phi_{\rm r} \approx 1/(1+\alpha_{\rm pr})$ (the melt regime), see Fig. 3 in the main article. For simplicity, we here derive the asymptotic forms of the volume fractions $\phi_{\rm r}$ and $\phi_{\rm p}$ for the case of $\alpha_{\rm pr} \approx 1$ (which is the case of $\epsilon < -1$). We derive the solution for

$$\phi_{\rm r} = \frac{1-\delta\phi_{\rm rm}}{2} \tag{S30}$$

$$\alpha_{\rm pr} = 1-\delta\alpha_{\rm pm} \tag{S31}$$

with $\delta\phi_{\rm rm} \ll 1$, $\delta\alpha_{\rm pm} \ll 1$, and $\phi_{\rm p} \ll 1$. With this approximation, eq. S20 is rewritten in the form

$$\frac{\Pi_{\rm ex}b^3}{k_{\rm B}T} = -1 - \log(1-2\phi_{\rm r}) - \frac{1}{4}\chi - 6(\sigma_{\rm in}b^2)^2. \tag{S32}$$

Supplementary Equation (S32) is derived by substituting Supplementary Equations (S30) and (S31), by expanding it in a power series of $\delta\phi_{\rm rm}$, $\delta\alpha_{\rm pm}$, and $\phi_{\rm p}$. We assumed that $\phi_{\rm p}$ is very small and neglected even the terms linear to $\phi_{\rm p}$. We also used the boundary condition $\Pi = \Pi_{\rm ex}$ for the planer geometry, see Supplementary Equation (S22) for $r_{\rm ex} \to \infty$. The volume fraction $\phi_{\rm r}$ of the FBRs of nascent pre-rRNAs is derived as

$$\phi_{\rm rm} \;=\; \frac{1}{2}\left(1 - \mathrm{e}^{-1-\frac{1}{4}\chi - \Pi_{\rm ex} b^3/(k_{\rm B}T)}\right).,\tag{S33}$$

by using Supplementary Equation (S32). To derive Supplementary Equation (S33), we further neglected the fourth term of Supplementary Equation (S32), which is usually smaller than the other terms of Supplementary Equation (S32), see Table 1 in the main article.

With the approximation, Supplementary Equations (S30) and (S30), Supplementary Equation (S15) is rewritten in the form

$$\frac{\mu_{\rm p}}{k_{\rm B}T} = \log\phi_{\rm p} - \chi - \log(1 - 2\phi_{\rm rm}).\tag{S34}$$

The volume fraction of RBPs thus has the form

$$\phi_{\rm pm} = \mathrm{e}^{\mu_{\rm p}/(k_{\rm B}T) - 1 + \frac{3}{4}\chi}.\tag{S35}$$

The volume fraction of solvent molecules has the form

$$\phi_{\rm s} = 1 - \phi_{\rm pm} - 2\phi_{\rm r} = \mathrm{e}^{-1-\chi/4}\left(1 - \mathrm{e}^{\mu_{\rm p}/(k_{\rm B}T)+\chi}\right).\tag{S36}$$

The fact that $\phi_{\rm s} > 0$ implies that the approximate forms, Supplementary Equations (S33) and (S34), are effective for $\chi + \mu_{\rm p}/(k_{\rm B}T) < 0$.

DFC regime

For cases in which the interaction parameter is larger than the negative of the chemical potential of RBPs, $\chi > -\mu_{\mathrm{p}}/(k_{\mathrm{B}}T)$, most space of the DFC layer is occupied by RBPs (DFC regime), see Fig. 3 in the main article. In this regime, we derive the volume fractions in the form

$$\phi_{\mathrm{p}} = 1 - \delta\phi_{\mathrm{pd}} \tag{S37}$$

$$\tag{S38}$$

for $\delta\phi_{\mathrm{pd}} \ll 1$ and $\phi_{\mathrm{r}} \ll 1$. With this approximation, the occupancy of the FBRs of nascent pre-rRNAs has an asymptotic form

$$\alpha_{\mathrm{pd}} = \frac{\mathrm{e}^{-(\epsilon-1)}}{1+\mathrm{e}^{-(\epsilon-1)}}. \tag{S39}$$

Supplementary Equation (S39) is derived by substituting Supplementary Equation (S37) into Supplemeentary Equation (S16), expanding it in a power series of $\delta\phi_{\mathrm{pd}}$, and neglecting higher order terms of these parameters.

In the DFC regime, Supplementary Equations (S15) and (S20) has an approximate form

$$\frac{\mu_{\mathrm{p}}}{k_{\mathrm{B}}T} = -\log(\delta\phi_{\mathrm{p}} - (1+\alpha_{\mathrm{pd}})\phi_{\mathrm{r}}) - 2\chi \tag{S40}$$

$$\frac{\Pi_{\mathrm{ex}}b^3}{k_{\mathrm{B}}T} = -\log(\delta\phi_{\mathrm{p}} - (1+\alpha_{\mathrm{pd}})\phi_{\mathrm{r}}) - \chi - \frac{3(\sigma_{\mathrm{in}}b^2)^2}{\phi_{\mathrm{r}}} \tag{S41}$$

where they are derived by substituting Supplementary Equation (S37) into Supplementary Equations (S15) and (S20), expanding them in a power series of $\delta\phi_{\mathrm{pd}}$ and ϕ_{r}, and neglecting the higher order terms of these parameters. We also used the boundary condition $\Pi = \Pi_{\mathrm{ex}}$ for the planer geometry, see Supplementary Equation (S22) for $r_{\mathrm{ex}} \to \infty$. Eliminating the term $\log(\delta\phi_{\mathrm{p}} - (1+\alpha_{\mathrm{pd}})\phi_{\mathrm{r}})$ in Supplementary Equation (S41) with Supplementary Equation (S40),

the relationship between the volume fraction $\phi_{\rm r}$ and the external pressure $\Pi_{\rm ex}$ is derived in the form

$$\frac{\Pi_{\rm ex} b^3}{k_{\rm B}T} = \chi + \frac{\mu_{\rm p}}{k_{\rm B}T} - \frac{3(\sigma_{\rm in} b^2)^2}{\phi_{\rm r}}. \tag{S42}$$

The volume fraction of the FBRs of nascent pre-rRNAs thus has the form

$$\phi_{\rm rd} = \frac{3(\sigma_{\rm in} b^2)^2}{\chi + (\mu_{\rm p} - \Pi_{\rm ex} b^3)/(k_{\rm B}T)}. \tag{S43}$$

Supplementary Note 4: Asymptotic form of spherical geometry

The local volume fractions of the FBRs of nascent pre-rRNA $\phi_{\rm r}(r)$ and RBPs $\phi_{\rm p}(r)$ are derived by using Supplementary Equations (S15), (S16), (S17), and (S22) for the spherical geometry. We derive the volume fractions, the free energy F of the system, and the radius $r_{\rm in}$ of FCs for $\alpha_{\rm p} \approx 1$ and $\chi > -\mu_{\rm p}/(k_{\rm B}T)$. The approximation, $\alpha_{\rm p} \approx 1$, is effective for $\epsilon - 1 - \mu_{\rm p}/(k_{\rm B}T) \ll -1$, where RBPs are bound to the FBRs of pre-rRNA even in a dilute solution. In this case, the layer composed of the FBRs of nascent pre-rRNAs can be a uniform DFC layer, a uniform melt layer, and a double layer of DFC and melt regions.

DFC layer

In the DFC regime, most volume of the layer composed of the FBRs of nascent pre-rRNAs is occupied by RBPs. We thus derive the solution of Supplementary Equations (S13), (S15), (S16), and (S22) in the form of

$$\phi_{\rm p}(r) = 1 - \delta\phi_{\rm p}(r), \tag{S44}$$

where $\delta\phi_{\rm p} \ll 1$ and $\phi_{\rm r} \ll 1$. In this case, Supplementary Equation (S15) has an approximate form

$$\frac{\mu_{\rm p}}{k_{\rm B}T} = -\log(\delta\phi_{\rm p}(r) - 2\phi_{\rm r}(r)) - 2\chi. \tag{S45}$$

Supplementary Equation (S45) is derived by substituting Supplementary Equation (S44), expanding it in a power series of $\delta\phi_{\rm p}(r)$ and $\phi_{\rm r}$, and neglecting the higher order terms with respect to $\delta\phi_{\rm p}(r)$ and $\phi_{\rm r}$. By using the same expansion and approximation, Supplementary Equation (S17) is rewritten in the form

$$\begin{aligned}\frac{\mu_{\rm r}}{k_{\rm B}T} &= -\frac{3}{2}\frac{(\sigma_{\rm in}b^2)^2}{\phi_{\rm r}^2(r)}\frac{r_{\rm in}^4}{r^4} - 2\log(\delta\phi_{\rm p} - 2\phi_{\rm r}) - 2 - 2\chi + \epsilon - \frac{\mu_{\rm p}}{k_{\rm B}T} \\ &= -\frac{3}{2}\frac{(\sigma_{\rm in}b^2)^2}{\phi_{\rm r}^2(r)}\frac{r_{\rm in}^4}{r^4} + \frac{\mu_{\rm p}}{k_{\rm B}T} + 2\chi + \epsilon - 2. \end{aligned} \tag{S46}$$

The last form of Supplementary Equation (S46) is derived by eliminating $\log(\delta\phi_{\rm p} - 2\phi_{\rm r})$ by using Supplementary Equation (S45). By solving Supplementary Equation (S46) with respect to $\phi_{\rm r}(r)$, the volume fraction $\phi_{\rm r}(r)$ of the FBRs of nascent pre-rRNAs is derived as

$$\phi_{\rm r}(r) = \sqrt{\frac{3}{2}}\frac{\sigma_{\rm in}b^2}{\sqrt{\Delta\mu_{\rm r}/(k_{\rm B}T)}}\frac{r_{\rm in}^2}{r^2}, \tag{S47}$$

where we introduced a parameter

$$\frac{\Delta\mu_{\rm r}}{k_{\rm B}T} = \frac{\mu_{\rm p}}{k_{\rm B}T} + 2\chi + \epsilon - 2 - \frac{\mu_{\rm r}}{k_{\rm B}T} \tag{S48}$$

to simplify the expression of Supplementary Equation (S47).

The osmotic pressure has an approximate form

$$\begin{aligned}\frac{\Pi b^3}{k_{\rm B}T} &= -\log(\delta\phi_{\rm p}(r) - 2\phi_{\rm r}(r)) - \chi - \frac{3(\sigma_{\rm in}b^2)^2}{\phi_{\rm r}(r_{\rm ex})}\frac{r_{\rm in}^4}{r_{\rm ex}^4} \\ &= \chi + \frac{\mu_{\rm p}}{k_{\rm B}T} - \frac{3(\sigma_{\rm in}b^2)^2}{\phi_{\rm r}(r)}\frac{r_{\rm in}^4}{r^4}, \end{aligned} \tag{S49}$$

where it is derived by substituting Supplementary Equation (S44) into Supplementary Equation (S20), expanding it in a power series of $\delta\phi_{\rm p}(r)$ and $\phi_{\rm r}(r)$, and neglecting the higher order terms with respect to $\delta\phi_{\rm p}(r)$ and $\phi_{\rm r}(r)$. The last form of Supplementary Equation (S49) is derived by eliminating $\log(\delta\phi_{\rm p}(r) - 2\phi_{\rm r}(r))$ by using Supplementary Equation (S46). By using Supplementary Equation (S22), the volume fraction $\phi_{\rm rex}$ ($= \phi_{\rm r}(r_{\rm ex})$) of the FBRs of nascent pre-rRNAs at $r = r_{\rm ex}$ is derived as

$$\phi_{\rm rex} = \frac{3(\sigma_{\rm in} b^2)^2}{\chi + (\mu_{\rm p} - \Pi_{\rm ex})/(k_{\rm B}T) - 2\gamma_{\rm p} b^3/r_{\rm ex}} \frac{r_{\rm in}^4}{r_{\rm ex}^4}. \tag{S50}$$

By using the boundary condition, Supplementary Equation (S50), the local volume fraction $\phi_{\rm r}(r)$ of the FBRs of nascent pre-rRNA has the form

$$\phi_{\rm r}(r) = \phi_{\rm rex} \frac{r_{\rm ex}^2}{r^2}. \tag{S51}$$

The chemical potential $\mu_{\rm r}$ has the form

$$\frac{\mu_{\rm r}}{k_{\rm B}T} = \frac{\mu_{\rm p}}{k_{\rm B}T} + 2\chi + \epsilon - 2 - \frac{1}{6}\frac{1}{(\sigma_{\rm in} b^2)^2}\left(\chi + \frac{\mu_{\rm p} - \Pi_{\rm ex}}{k_{\rm B}T} - \frac{2\gamma_{\rm p} b^3}{r_{\rm ex}}\right)\frac{r_{\rm ex}^4}{r_{\rm in}^4}. \tag{S52}$$

Supplementary Equations (S50), (S51), and (S52) are effective for cases in which the layer of the FBRs of nascent pre-rRNAs is a double layer of the DFC and melt phases if the DFC layer interfaces with GC at $r = r_{\rm ex}$.

The external radius $r_{\rm ex}$ is derived by using the relationship

$$\frac{r_{\rm in}^2}{r_{\rm ex}^2}\left(\frac{r_{\rm ex}}{r_{\rm in}} - 1\right) = \frac{N_{\rm r} b}{3(\sigma_{\rm in} b^2) r_{\rm in}}\left(\chi + \frac{\mu_{\rm p} - \Pi_{\rm ex}}{k_{\rm B}T} - \frac{2\gamma_{\rm p} b^3}{r_{\rm ex}}\right), \tag{S53}$$

where it is derived by substituting Supplementary Equation (S51) (and Supplementary Equation (S50)) into Supplementary Equation (S4). Indeed, Supplementary Equation (S53) is a quadratic equation and the ratio $r_{\rm ex}/r_{\rm in}$ can be derived analytically.

The free energy density of the layer of the FBRs of nascent pre-rRNAs has an approximate form

$$\begin{aligned}\frac{f_{\rm d}}{k_{\rm B}t} &= \frac{3}{2}\frac{(\sigma_{\rm in}b^2)^2}{\phi_{\rm r}(r)}\frac{r_{\rm in}^4}{r^4} - \left(\chi + \frac{\mu_{\rm p}-\Pi_{\rm ex}}{k_{\rm B}T}\right) \\ &= \frac{1}{2}\left(\chi + \frac{\mu_{\rm p}-\Pi_{\rm ex}}{k_{\rm B}T} - 2\gamma_{\rm p}b^3/r_{\rm ex}\right)\frac{r_{\rm ex}^2}{r^2} - \left(\chi + \frac{\mu_{\rm p}-\Pi_{\rm ex}}{k_{\rm B}T}\right). \qquad \text{(S54)}\end{aligned}$$

Supplementary Equation (S54) is derived by substituting Supplementary Equations (S44), expanding it in a power series of $\delta\phi_{\rm p}$ and $\phi_{\rm r}$, and neglecting the higher order terms of $\delta\phi_{\rm p}$ and $\phi_{\rm r}$. We also used Supplementary Equation (S51) to derive the last form of Supplementary Equation (S54). By using Supplementary Equation (S54), the free energy of the system is derived as

$$\begin{aligned}\frac{Fb^3}{3V_{\rm m}k_{\rm B}T} &= \frac{1}{6}\left(\chi + \frac{\mu_{\rm p}-\Pi_{\rm ex}}{k_{\rm B}T}\right)\left[\left(\frac{r_{\rm ex}}{r_{\rm in}}\right)^3 - 3\left(\frac{r_{\rm ex}}{r_{\rm in}}\right)^2 + 2\right] + \frac{\gamma_{\rm p}b^3}{k_{\rm B}Tr_{\rm in}}\left(1 + \frac{r_{\rm ex}}{r_{\rm in}}\right) \\ &\quad + \zeta\left(\epsilon + 2\chi + \frac{\mu_{\rm p}}{k_{\rm B}T} - 2\right), \qquad \text{(S55)}\end{aligned}$$

where we used

$$\zeta = N_{\rm r}\frac{\sigma_{\rm in}b^3}{r_{\rm in}}. \qquad \text{(S56)}$$

ζ is proportional to the transcription rate. The free energy, Supplementary Equation (S55), depends on the size of FCs via the ratio $r_{\rm ex}/r_{\rm in}$ and $\sigma_{\rm in}(\propto r_{\rm in})$, see also Supplementary Equation (S53).

For $\gamma_{\rm p}b^3/r_{\rm in} \ll \chi + (\mu_{\rm p}-\Pi_{\rm ex})/(k_{\rm B}T)$, the first term of Supplementary Equation (S55) dominates the second term of this equation. In this case, the first derivative of the free energy with respect to the raio $r_{\rm ex}/\text{in}$ is zero at the minimum of the free energy,

$$\frac{\partial}{\partial(r_{\rm ex}/r_{\rm in})}\left(\frac{Fb^3}{3V_{\rm m}k_{\rm B}T}\right) \simeq \frac{1}{2}\left(\chi + \frac{\mu_{\rm p}-\Pi_{\rm ex}}{k_{\rm B}T}\right)\frac{r_{\rm ex}}{r_{\rm in}}\left(\frac{r_{\rm ex}}{r_{\rm in}} - 2\right) = 0, \qquad \text{(S57)}$$

This leads to

$$\frac{r_{\rm ex}}{r_{\rm in}} = 2. \tag{S58}$$

By substituting Supplementary Equation (S58) into Supplementary Equation (S53), the radius of FCs is derived as

$$\frac{r_{\rm in}}{N_{\rm r}b} = \frac{\sigma_{\rm in}b^2}{\zeta} = \frac{2}{\sqrt{3}}\left(\chi + \frac{\mu_{\rm p} - \Pi_{\rm ex}}{k_{\rm B}T}\right)^{1/2}\zeta^{-1/2}, \tag{S59}$$

implying that the radius of FCs is proportional to the inverse of the square root of the transcription rate.

Melt layer

For cases in which the FBRs of pre-rRNAs form a uniform melt layer, the volume fraction $\phi_{\rm r}$ of the FBRs of pre-rRNAs is $\approx 1/2$. We therefore derive the volume fractions in the forms

$$\phi_{\rm r} = \frac{1}{2}(1 - \delta\phi_{\rm r}), \tag{S60}$$

where $\phi_{\rm p} \ll 1$ and $\delta\phi_{\rm r} \ll 1$. We substitute Supplementary Equation (S60) into Supplementary Equations (S15), (S20), (S17), expanding these equations in a power series of $\phi_{\rm p}$ and $\delta\phi_{\rm r}$, and neglecting the higher order terms with respect to $\phi_{\rm p}$ and $\delta\phi_{\rm r}$,

$$\frac{\mu_{\rm p}}{k_{\rm B}T} = \log\phi_{\rm p} - \log(\delta\phi_{\rm r} - \delta\phi_{\rm p}) - \chi \tag{S61}$$

$$\frac{\mu_{\rm r}}{k_{\rm B}T} = -6(\sigma(r)b^2)^2 - 2\log(\delta\phi_{\rm r} - \delta\phi_{\rm p}) - 2 - \chi + \epsilon - \frac{\mu_{\rm p}}{k_{\rm B}T} \tag{S62}$$

$$\frac{\Pi b^3}{k_{\rm B}T} = -\log(\delta\phi_{\rm r} - \phi_{\rm p}) - 1 - \frac{1}{4}\chi - 6(\sigma(r)b^2)^2. \tag{S63}$$

The osmotic pressure Π is derived by eliminating $\log(\delta\phi_{\rm r} - \phi_{\rm p})$ in Supplementary Equation (S63) by using Supplementary Equation (S62). This leads to the form

$$\frac{\Pi b^3}{k_{\rm B}T} = \frac{1}{2}\left(\frac{\mu_{\rm r}+\mu_{\rm p}}{k_{\rm B}T} - \epsilon\right) + \frac{1}{4}\chi - 3(\sigma(r)b^2)^2. \tag{S64}$$

By substituting Supplementary Equation (S62) into Supplementary Equation (S4), the ratio $r_{\rm ex}/r_{\rm in}$ is derived as

$$\frac{r_{\rm ex}}{r_{\rm in}} = (1+6\zeta)^{1/3}, \tag{S65}$$

where ζ is given by Supplementary Equation (S56). The ratio $r_{\rm ex}/r_{\rm in}$ thus does not depend on the radius of FCs. The free energy has an approximate form

$$\frac{Fb^3}{3V_{\rm m}k_{\rm B}T} = -\frac{1}{6}\left(\frac{1}{2}\chi + \frac{\mu_{\rm p}}{k_{\rm B}T}\right)\left(\frac{r_{\rm ex}^3}{r_{\rm in}^3} - 1\right) + 3(\sigma_{\rm in}b^2)^2\left(1 - \frac{r_{\rm in}}{r_{\rm ex}}\right) + \frac{1}{2}\frac{\gamma_{\rm p}b^3}{k_{\rm B}Tr_{\rm in}}\left(1 + \frac{r_{\rm ex}^2}{r_{\rm in}^2}\right), \tag{S66}$$

where it is derived by using $\phi_{\rm r} \approx 1/2$ and $\phi_{\rm p} \simeq 1 - 2\phi_{\rm r} \ll 1$ to Supplementary Equation (S1). The derivative of the free energy with respect to $\sigma_{\rm in}b^2$ is zero at the minimum of the free energy,

$$\frac{\partial}{\partial(\sigma_{\rm in}b^2)}\left(\frac{Fb^3}{3V_{\rm m}k_{\rm B}T}\right) = 6(\sigma_{\rm in}b^2)\left(1 - \frac{r_{\rm in}}{r_{\rm ex}}\right) - \frac{1}{2}\frac{\gamma_{\rm p}b^2\zeta}{N_{\rm r}k_{\rm B}T}\frac{1}{(\sigma_{\rm in}b^2)^2}\left(1 + \frac{r_{\rm ex}^2}{r_{\rm in}^2}\right) = 0. \tag{S67}$$

The radius of FCs thus has the form

$$\frac{r_{\rm in}}{N_{\rm r}b} = \left(\frac{\gamma_{\rm p}b^2}{12N_{\rm r}k_{\rm B}T}\right)^{1/3}\zeta^{-2/3}\left(\frac{1 + r_{\rm ex}^2/r_{\rm in}^2}{1 - r_{\rm in}/r_{\rm ex}}\right)^{1/3}, \tag{S68}$$

see Supplementary Equation (S65) for the dependence of $r_{\rm ex}/r_{\rm in}$ on ζ. For $\zeta \ll 1$, eq. S68 has an approximate form

$$\frac{r_{\rm in}}{N_{\rm r}b} \simeq \left(\frac{\gamma_{\rm p}b^2}{12N_{\rm r}k_{\rm B}T}\right)^{1/3}\zeta^{-1}. \tag{S69}$$

Double layer of DFC and melt

In some cases, the layer occupied by the FBRs of nascent pre-rRNAs is composed of a double layer of a melt phase and a DFC phase. The chemical potentials, μ_p and μ_r, and the osmotic pressure Π is continuous at the interface between the two layers. The osmotic pressure increases with increasing the radial coordinate r in the DFC layer, see Supplementary Equation (S49), while the osmotic pressure is almost constant in the melt layer, see Supplementary Equation (S64) (where the third term of eq. S64 is smaller than the other terms in this equation and is neglected). This implies that the DFC layer is at the interface with the GC and the melt layer at the interface with the FC. The osmotic pressure in the DFC layer has the form

$$\frac{\Pi b^3}{k_\mathrm{B}T} = \chi + \frac{\mu_\mathrm{p}}{k_\mathrm{B}T} - \left(\chi + \frac{\mu_\mathrm{p} - \Pi_\mathrm{ex} b^3}{k_\mathrm{B}T} - \frac{2\gamma_\mathrm{ex} b^3}{k_\mathrm{B}T r_\mathrm{ex}}\right) \frac{r_\mathrm{ex}^2}{r^2}, \tag{S70}$$

where it is derived by substituting Supplementary Equation (S51) into Supplementary Equation (S49). The osmotic pressure in the melt layer has the form

$$\frac{\Pi b^3}{k_\mathrm{B}T} = -\frac{1}{12(\sigma_\mathrm{in} b^2)^2} \left(\chi + \frac{\mu_\mathrm{p} - \Pi_\mathrm{ex} b^3}{k_\mathrm{B}T} - \frac{2\gamma_\mathrm{ex} b^3}{k_\mathrm{B}T r_\mathrm{ex}}\right)^2 \frac{r_\mathrm{ex}^4}{r_\mathrm{in}^4} + \frac{\mu_\mathrm{p}}{k_\mathrm{B}T} + \frac{5}{4}\chi - 1, \tag{S71}$$

where it is derived by substituting the chemical potential

$$\frac{\mu_\mathrm{r}}{k_\mathrm{B}T} = -\frac{3}{2} \frac{(\sigma_\mathrm{in} b^2)^2}{\phi_\mathrm{rex}^2} \frac{r_\mathrm{in}^4}{r_\mathrm{ex}^4} + \frac{\mu_\mathrm{p}}{k_\mathrm{B}T} + 2\chi + \epsilon - 2. \tag{S72}$$

in Supplementary Equation (S64) and neglected the third term of Supplementary Equation (S64). Supplementary Equation (S72) is derived by substituting Supplementary Equation (S51) into Supplementary Equation (S46) and is effective for cases in which the DFC layer interfaces with the GC (because Supplementary Equation (S64) is imposed at $r = r_\mathrm{ex}$). The radical coordinate r_i at the interface between the DFC and melt layer is derived by equating

Supplementary Equations (S70) and (S71),

$$\frac{r_{\rm i}^2}{r_{\rm in}^2} = \frac{\left(\chi + \frac{\mu_{\rm p} - \Pi_{\rm ex} b^3}{k_{\rm B}T} - \frac{2\gamma_{\rm ex} b^3}{k_{\rm B}T r_{\rm ex}}\right) \frac{r_{\rm ex}^2}{r_{\rm in}^2}}{1 - \frac{1}{4}\chi + \frac{1}{12(\sigma_{\rm in} b^2)^2}\left(\chi + \frac{\mu_{\rm p} - \Pi_{\rm ex} b^3}{k_{\rm B}T} - \frac{2\gamma_{\rm ex} b^3}{k_{\rm B}T r_{\rm ex}}\right)^2 \frac{r_{\rm ex}^4}{r_{\rm in}^4}}. \tag{S73}$$

The volume fraction $\phi_{\rm r}$ of the units of nascent pre-rRNAs is given by eq. S51 in the DFC layer ($r_{\rm i} < r < r_{\rm ex}$) and is $\approx 1/2$ in the melt layer ($r_{\rm in} < r < r_{\rm i}$),

$$\phi_{\rm r}(r) = \begin{cases} \frac{1}{2} & (r_{\rm in} < r < r_{\rm i}) \\ \phi_{\rm rex} \frac{r_{\rm ex}^2}{r^2} & (r_{\rm i} < r < r_{\rm ex}) \end{cases} \tag{S74}$$

A relationship

$$\frac{3(\sigma_{\rm in} b^2)^2}{\chi + \frac{\mu_{\rm p} - \Pi_{\rm ex} b^3}{k_{\rm B}T} - \frac{2\gamma_{\rm ex} b^3}{k_{\rm B}T r_{\rm ex}}} \frac{r_{\rm in}^2}{r_{\rm ex}^2}\left(\frac{r_{\rm ex}}{r_{\rm in}} - \frac{r_{\rm i}}{r_{\rm in}}\right) + \frac{1}{6}\left(\frac{r_{\rm i}^3}{r_{\rm in}^3} - 1\right) = \zeta. \tag{S75}$$

is derived by substituting Supplementary Equation (S74) into Supplementary Equation (S4). By using Supplementary Equations (S73) and (S75), one can derive the ratio $r_{\rm i}/r_{\rm in}$ and $r_{\rm ex}/r_{\rm in}$ as a function of $\sigma_{\rm in} b^2$ ($\propto r_{\rm in}$). The free energy of the system is derived as

$$\begin{aligned} \frac{F b^3}{3 k_{\rm B} T V_{\rm m}} &= \frac{1}{6}\left(\chi + \frac{\mu_{\rm p} - \Pi_{\rm ex} b^3}{k_{\rm B}T}\right)\left(\frac{r_{\rm ex}^3}{r_{\rm in}^3} - 3\frac{r_{\rm ex}^2}{r_{\rm in}^2}\frac{r_{\rm i}}{r_{\rm in}} + 2\frac{r_{\rm i}^3}{r_{\rm in}^3}\right) + \frac{\gamma_{\rm p} b^3}{k_{\rm B}T r_{\rm in}}\left(\frac{1}{2} + \frac{r_{\rm ex} r_{\rm i}}{r_{\rm in}^2}\right) + \frac{\gamma_{\rm p} b^3}{k_{\rm B}T r_{\rm in}}\frac{1}{2}\frac{r_{\rm i}^2}{r_{\rm in}^2} \\ &\quad - \frac{1}{6}\left(\frac{1}{2}\chi + \frac{\mu_{\rm p}}{k_{\rm B}T} - \frac{2\Pi_{\rm ex} b^3}{k_{\rm B}T} - \epsilon\right)\left(\frac{r_{\rm i}^3}{r_{\rm in}^3} - 1\right) \\ &\quad + \frac{3(\sigma_{\rm in} b^2)^2}{\chi + \frac{\mu_{\rm p} - \Pi_{\rm ex} b^3}{k_{\rm B}T} - \frac{2\gamma_{\rm ex} b^3}{k_{\rm B}T r_{\rm ex}}}\left(\epsilon + 2\chi + \frac{\mu_{\rm p}}{k_{\rm B}T} - 2\right)\frac{r_{\rm in}^2}{r_{\rm ex}^2}\left(\frac{r_{\rm ex}}{r_{\rm in}} - \frac{r_{\rm i}}{r_{\rm in}}\right). \end{aligned} \tag{S76}$$

Supplementary Note 5: Elastic free energy of nascent RNA transcripts

In the main article, we use an extension of the theory of polymer brush to treat nascent pre-rRNA transcripts. The Alexander approximation assumes that the volume fraction of

nascent pre-rRNA units is uniform for nascent pre-rRNA transcripts on a planer surface.[9,10] With this approximation, the free energy per unit area of nascent pre-rRNA transcripts on a planer surface has the form

$$\frac{F_{\mathrm{bru}}}{k_{\mathrm{B}}T} = \frac{3}{2}\frac{\sigma h^2}{N_{\mathrm{r}}b^2} + v\sigma N_{\mathrm{r}}\frac{\sigma N_{\mathrm{r}}}{h}. \tag{S77}$$

This free energy is a function of the height h of the nascent pre-rRNA transcripts. The first term of Supplementary Equation (S77) is the elastic free energy of nascent pre-rRNA transcripts and the second term of Supplementary Equation (S77) is the free energy due to the interactions between nascent pre-rRNA units. k_{B} is the Boltzmann constant and T is the absolute temperature. N_{r} is the number of units in a nascent pre-rRNA transcript and b is the length of each unit. σ is the surface density of nascent pre-rRNA transcripts. v is the excluded volume that represents the magnitude of the interactions between nascent pre-rRNA transcripts and has a relationship $v = b^3(2-\chi)$ with the interaction parameter χ introduced in eq. (16) in the main article (the difference from the usual relationship $b^3(1-2\chi)/2$ is due to the volume of RBPs bound to nascent pre-rRNA transcripts. Supplementary Equation (S77) is rewritten in the form

$$\frac{F_{\mathrm{bru}}}{k_{\mathrm{B}}T} = \frac{3}{2}\frac{b^4\sigma^3 N_{\mathrm{r}}}{\phi_{\mathrm{r}}^2} + \frac{v}{b^3}\sigma N_{\mathrm{r}}\phi_{\mathrm{r}} \tag{S78}$$

by using the form of the volume fraction of nascent pre-rRNA units

$$\phi_{\mathrm{r}} = \frac{b^3\sigma N_{\mathrm{r}}}{h}. \tag{S79}$$

Minimizing Supplementary Equation (S77) with respect to h leads to

$$h = N_{\mathrm{r}}b\left(\frac{\sigma v}{3b}\right)^{1/3}. \tag{S80}$$

The volume fraction of nascent pre-rRNA units is derived as

$$\phi_{\mathrm{r}} \;=\; \left(\frac{3\sigma^2 b^7}{v}\right)^{1/3} \tag{S81}$$

by substituting Supplementary Equation (S80) into Supplementary Equation (S79) or by minimizing Supplementary Equation (S78) with respect to ϕ_{r}.

Supplementary Equation (S77) is rewritten in the following form

$$\frac{F_{\mathrm{bru}}}{k_{\mathrm{B}}T} \;=\; \frac{\sigma N_{\mathrm{r}}}{g}\left[\frac{3}{2}\frac{\xi^2}{gb^2} + vg\frac{g}{\xi^3}\right]. \tag{S82}$$

With this expression, we divide nascent pre-rRNA transcripts into blobs of size ξ ($\equiv \sigma^{-1/2}$), which the average distance between grafting points (to which nascent pre-rRNA transcripts are end-grafted) at the surface. g is the number of nascent pre-rRNA units in each blob. The first term in the square bracket in Supplementary Equation (S82) is the elastic free energy of the subchain in a blob and the second term in the square bracket is the free energy due to the interactions between nascent pre-rRNA units in the blob. The prefactor N_{r}/g is the number of blobs in each nascent pre-rRNA transcript. Supplementary Equation (S82) returns to Supplementary Equation (S78) by using the form of the local volume fraction of nascent pre-rRNA units

$$\phi_{\mathrm{r}} = \frac{b^3 g}{R^3}. \tag{S83}$$

Now we extend the above formalism to nascent pre-rRNA transcripts on a spherical surface, see Fig. 1B in the main article. The size of each blob is

$$\xi = \frac{r}{\sigma^{1/2} r_{\mathrm{in}}} \tag{S84}$$

due to the fact that the spherical surface at the distance r from the center of this surface is

occupied by $4\pi r_{\text{in}}^2\sigma$ transcripts,

$$4\pi r^2 = (4\pi r_{\text{in}}^2\sigma)\xi^2. \tag{S85}$$

The number of blobs in the spherical shell of thickness dr at the distance r from the center is $4\pi r^2 dr/\xi^3$. Supplementary Equation (S82) is extended to

$$\frac{F_{\text{bru}}}{k_{\text{B}}T} = \int_{r_{\text{in}}}^{r_{\text{ex}}} \frac{4\pi r^2 dr}{\xi^3}\left[\frac{3}{2}\frac{\xi^2}{gb^2} + vg\frac{g}{\xi^3}\right], \tag{S86}$$

where it is along with the spirit of the Daoud-Cotton theory.[11] Supplementary Equation (S86) is rewritten in the form

$$\frac{F_{\text{bru}}}{k_{\text{B}}T} = \int_{r_{\text{in}}}^{r_{\text{ex}}} \frac{4\pi r^2 dr}{b^3}\left[\frac{3}{2}\frac{b^4\sigma^2 r_{\text{in}}^4}{r^4}\frac{1}{\phi_{\text{r}}} + \frac{v}{b^3}\phi_{\text{r}}^2\right] \tag{S87}$$

by using Supplementary Equation (S83) and $R = \sigma^{-1/2}$. The first term in the square bracket of Supplementary Equation (S87) is the elastic free energy of the chain subsection in a blob of nascent pre-rRNA transcripts and the second term in the square bracket of Supplementary Equation (S87) is the free energy due to the interactions between nascent pre-rRNA units in the blob. We thus used the first term of Supplementary Equation (S87) for the elastic free energy, see eq. (14) in the main article.

Supplementary Discussion

Contribution of DNA to FC size

Not only the terminal regions of nascent pre-rRNAs, but also rDNAs are localized at the surfaces of FCs. One may think that rDNAs also contribute to the suppression of the growth of FCs. The rDNA is bound to the surface of FCs and thus bent to the curvature of the

surface. With these contributions, the free energy has the form

$$F = \frac{3\gamma_{\rm in} V_{\rm m}}{r_{\rm in}} + k_{\rm B}T \frac{l_{\rm DNA} L}{2 r_{\rm in}^2} - k_{\rm B}T \epsilon_{\rm DNA} \quad \text{(S88)}$$

This free energy is composed of the surface free energy due to the surface tension $\gamma_{\rm in}$ between FC and DFC (the first term), the elastic free energy due to the bending of rDNA (the second term), and is the free energy due to the binding of rDNA to the surface of FCs (the third term). $k_{\rm B}T\epsilon_{\rm DNA}$ is the free energy due to the binding of rDNA, $l_{\rm DNA}$ ($\approx$ 50 nm) is the persistence length of DNA, and $L_{\rm DNA}$ is the total length of rDNA bound at the surface of FCs. Supplementary Equation (S88) is derived by using the fact that the sum of the volume $V_{\rm m}$ of FCs is constant. Both the surface free energy and the bending free energy of rDNA decreases with increasing the radius $r_{\rm in}$ of FCs (see the first and second terms in Supplementary Equation (S88)), while the binding free energy is constant (see the third term in Supplementary Equation (S88)), implying that rDNA itself does not have a function to suppress the fusion of FCs. If we assume that the radius of FCs is $r_{\rm in} \sim 100$ nm, the first term is estimated as 9×10^{-19} J (by using $\gamma_{\rm in} \sim 1 \times 10^{-6}$ N/m), while the second term is estimated as 6×10^{-20}J (by using the fact that there are typically 10 FCs per nucleolus[1] and the persistence length of DNA is $l_{\rm DNA} \approx 50$ nm in the physiological salt concentration[12]), see also Table 2. This estimate implies that the first term of Supplementary Equation (S88) dominates the second term of Supplementary Equation (S88) for the FC radius $r_{\rm in}$ treated in our theory, $r_{\rm in} \leq 100$ nm and experiments. The above estimate does not take into account the fact that a stretch of DNA can be associated with multiple FCs. It is well-known in the colloid science that the polymers bridging between liquid droplets rather drive the coalescence of these droplets due to their entropic elasticity.[13] The above estimate suggests that rDNA is not likely to play a significant role in the size control of FCs, but a separate study is necessary to fully pursue it.

Relationship with conventional picture of ribosome biogenesis

The key assumption of our theory is that the terminal regions of nascent pre-rRNAs span between the top to the bottom of the DFC layer. This is motivated by a recent experiment that the upstream half of ETSs are localized at the DFC layer and the downstream half is localized at the FC.[14] The stretching of the terminal regions results from the multivalent interactions between FBLs and thus our theory should be tested in the condition of high FBL volume fraction. In the conventional picture, the DFC layer is composed of 5' ETSs that are already cleaved and folded by RNA processing. Such diffusive molecules tend to make the interaction between condensates attractive (the depletion interaction),[4] however, the non-equilibrium nature of the transcription may change the situation. We envisage a future theoretical study to predict the stable size of FCs with the conventional picture to check if the latter agrees with existing experiments, including ours.

Rheology of DFC layer

In our model, we focused on FBLs because these RBPs contribute most significantly to the localization of the terminal regions of pre-rRNAs to the DFC layer.[14] FRAP experiments on the condensates of FBLs reconstituted in vitro suggest that these condensates are thixotropic (and probably also be viscoelastic) and become gels in a long timescale.[5] Indeed, recent experiments showed that long non-coding RNA SLERT facilitates the transition of the RNA helicase DDX21 to the closed conformation and ensures the fluidity of proteins in the DFC layer.[15] This mechanism ensures the validity of our assumption that unbound FBLs can diffuse freely in the DFC layer, although we did not take into account these factors explicitly.

References

(1) Lafontaine, D.; Riback, J.; Bascetin, R.; Brangwynne, C. The nucleolus as a mutiphase liquid condensate. *Nat. Rev. Mol. Cell Biol.* **2021**, *22*, 165–182.

(2) Roth, E.; Azaria, A.; Girshevitz, O.; Bitler, A.; Garini, Y. Measuring the conformation and persistence length of single-strand DNA using a DNA origami structure. *Nano. Lett.* **2018**, *18*, 6703–6709.

(3) de Gennes, P. G.; Brochard-Wyart, F.; ; Quéré, D. *Wetting phenomena: Drops, Bubbles, Pearls, Waves.*; Springer: NY, US., 2004.

(4) Safran, S. *Statistical Thermodynamics of Surfaces, Interfaces, and Membranes*; Westview Press: CO, US., 2003.

(5) Feric, M.; Vaidya, N.; Harmon, T.; Mitrea, D.; Zhu, L.; Richardson, T.; Kriwacki, R.; Pappu, R.; Brangwynne, C. Coexisting Liquid Phases Underlie Nucleolar Subcompartments. *Cell* **2016**, *165*, 1686–1697.

(6) Stasevich, T.; Hayashi-Takanaka, Y.; Sato, Y.; Maehara, K.; Ohkawa, Y.; Sakata-Sogawa, K.; Tokunaga, M.; Nagase, T.; Nozaki, N.; McNally, J.; Kimura, H. Regulation of RNA polymerase II activation by histone acetylation in single living cells. *Nature* **2014**, *516*, 272–275.

(7) Dundr, M.; Hoffmann-Rohrer, U.; Hu, Q.; Grummt, I.; Rothblum, L.; Phair, R.; Misteli, T. Mammalian RNA Polymerase in Vivo. *Science* **2002**, *298*, 1623–1626.

(8) Stefanovsky, V.; Langlois, F.; Gagnon-Kugler, T.; Rothblum, L. I.; Moss, T. Growth factor signaling regulates elongation of RNA polymerase I transcription in mammals via UBF phosphorylation and r-chromatin remodeling. *Mol. Cell* **2006**, *21*, 629–639.

(9) Alexander, S. Adsorption of chain molecules with a polar head a scaling description. *J. Phys. France* **1980**, *38*, 983–987.

(10) de Gennes, P. Conformation of Polymers Attached to an Interface. *Macromolecules* **1980**, *13*, 1069–1075.

(11) Daoud, M.; Cotton, J. Star shaped polymers: a model for the conformation and its concentration dependence. *J. Phys. France* **1982**, *43*, 531–538.

(12) Marko, J.; Siggia, E. Stretching DNA. *Macromolecules* **1995**, *28*, 8759–8770.

(13) Doi, M. *Soft Matter Physics*; Oxford University Press: Oxford, UK., 2013.

(14) Yao, R.; Xu, G.; Wang, Y.; Shan, L.; Luan, P.; Wang, Y.; Wu, M.; Yang, L.; Xing, Y.; Yang, L.; Chen, L. Nascent Pre-rRNA Sorting via Phase Separation Drives the Assembly of Dense Fibrillar Components in the Human Nucleolus. *Mol. Cell* **2019**, *76*, 767–783.

(15) Wu, M.; Xu, G.; Han, C.; Luan, P.; Xing, Y.; Nan, F.; Yang, L.; Huang, Y.; Yang, Z.; Shan, L.; Yang, L.; Liu, J.; Chen, L. lncRNA SLERT controls phase separation of FC/DFCs to facilitate Pol I transcription. *Science* **2021**, *373*, 547–555.
